# Digital twins in dermatology, current status, and the road ahead

**DOI:** 10.1038/s41746-024-01220-7

**Published:** 2024-08-26

**Authors:** Hossein Akbarialiabad, Amirmohammad Pasdar, Dédée F. Murrell

**Affiliations:** 1 Faculty of Medicine, UNSW Medicine, University of New South Wales, Sydney, NSW Australia; 2 School of Computing and Information Systems, University of Melbourne, Melbourne, VIC Australia; 3 Department of Dermatology, St George Hospital, Sydney, NSW Australia

**Keywords:** Medical research, Clinical trial design

## Abstract

Digital twins, innovative virtual models synthesizing real-time biological, environmental, and lifestyle data, herald a new era in personalized medicine, particularly dermatology. These models, integrating medical-purpose Internet of Things (IoT) devices, deep and digital phenotyping, and advanced artificial intelligence (AI), offer unprecedented precision in simulating real-world physical conditions and health outcomes. Originating in aerospace and manufacturing for system behavior prediction, their application in healthcare signifies a paradigm shift towards patient-specific care pathways. In dermatology, digital twins promise enhanced diagnostic accuracy, optimized treatment plans, and improved patient monitoring by accommodating the unique complexities of skin conditions. However, a comprehensive review across PubMed, Embase, Web of Science, Cochrane, and Scopus until February 5th, 2024, underscores a significant research gap; no direct studies on digital twins’ application in dermatology is identified. This gap signals challenges, including the intricate nature of skin diseases, ethical and privacy concerns, and the necessity for specialized algorithms. Overcoming these barriers through interdisciplinary efforts and focused research is essential for realizing digital twins’ potential in dermatology. This study advocates for a proactive exploration of digital twins, emphasizing the need for a tailored approach to dermatological care that is as personalized as the patients themselves.

## Introduction

Health digital twins represent a pioneering leap in personalized medicine, embodying virtual replicas of individual patient health that merge comprehensive biological, environmental, and lifestyle data. These sophisticated models simulate real-world physical conditions within a digital framework, enabling physicians to predict health outcomes, optimize treatment plans, and monitor disease progression in real-time^1,2^. Initially conceptualized for aerospace and manufacturing to predict system behaviors and maintenance needs, the application of digital twin technology in healthcare marks a significant evolution, offering unprecedented insights into patient-specific care pathways^2–5^. In medicine, digital twin technology has emerged as a frontier in personalizing patient care, with applications involving cardiology, neurology, surgery, and oncology, demonstrating its ability to enhance diagnostic accuracy, optimize treatment plans, and improve patient outcomes^6–9^.

Digital twins for health are achieved through the seamless integration of medical-purpose Internet of Things (IoT) devices^10–13^, deep phenotyping^14–16^, digital phenotyping^17–19^,and artificial intelligence (AI) for disease progression simulation and treatment evaluation^20,21^. IoT’s role is pivotal, providing continuous health data, while deep phenotyping uncovers genetic and molecular insights crucial for understanding individual health risks and conditions. Digital phenotyping further adds to this personalized approach by including environmental and lifestyle factors, enriching the patient’s health profile for more holistic care strategies. AI, the core analytical engine, leverages this data to offer predictive insights and personalized treatment pathways. Additionally, integrating cyber-physical systems^6,22^ ensures that digital twins can adapt in real-time, enhancing the precision and effectiveness of dermatological care.

**Fig. 1:**
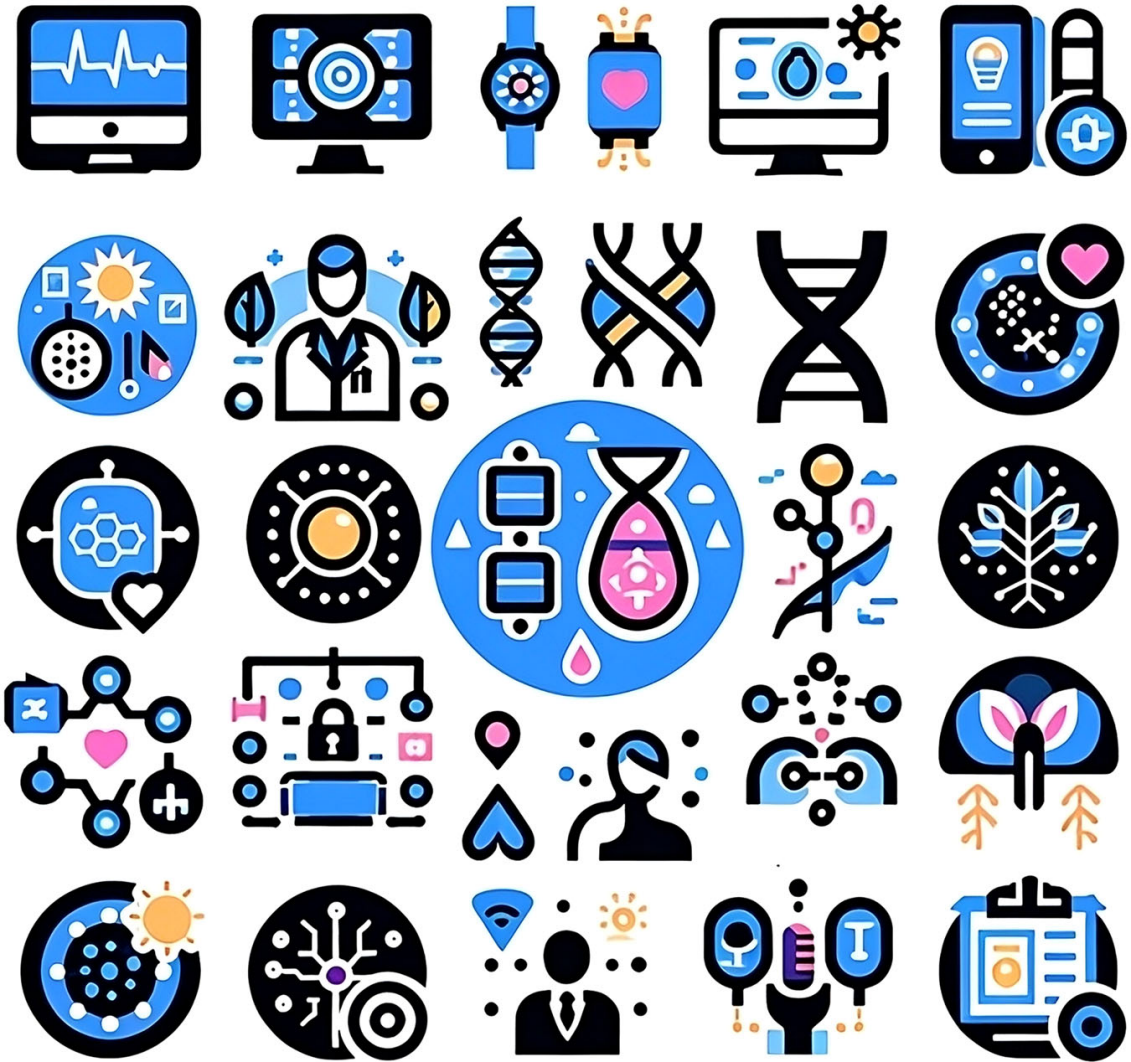
Elements of digital twins in dermatology. This figure illustrates the various components and technologies involved in the concept of digital twins in dermatology. It showcases the integration of IoT devices, deep phenotyping, digital phenotyping, and artificial intelligence. The visual representation emphasizes the combination of real-time biological, environmental, and lifestyle data to create sophisticated virtual models that simulate real-world conditions and predict health outcomes. The diagram highlights the potential for personalized care, enhanced diagnostic accuracy, and optimized treatment plans in dermatology, illustrating the interdisciplinary nature of this innovative technology.

The field of dermatology is experiencing a remarkable evolution, driven by the digital revolution that has begun to permeate medical science^23,24^. In recent years, the burgeoning interest in digital dermatology has been marked by the emergence of multiple AI applications, each promising to enhance diagnostic accuracy, treatment personalization, and patient monitoring^20^. Among the interdisciplinary innovations garnering attention within the dermatology community, the digital twin concept stands out as a promising frontier. This technology, by merging deep phenotyping^14^ with digital phenotyping^17,18^ and the IoT^25^, offers a comprehensive and dynamic representation of the patient’s health and skin condition, setting the stage for a new era of personalized dermatological care^7^. This approach is particularly pertinent given the skin’s status as the body’s largest organ^26^, subject to various conditions influenced by internal and external factors (Fig. 1).

**Fig. 2:**
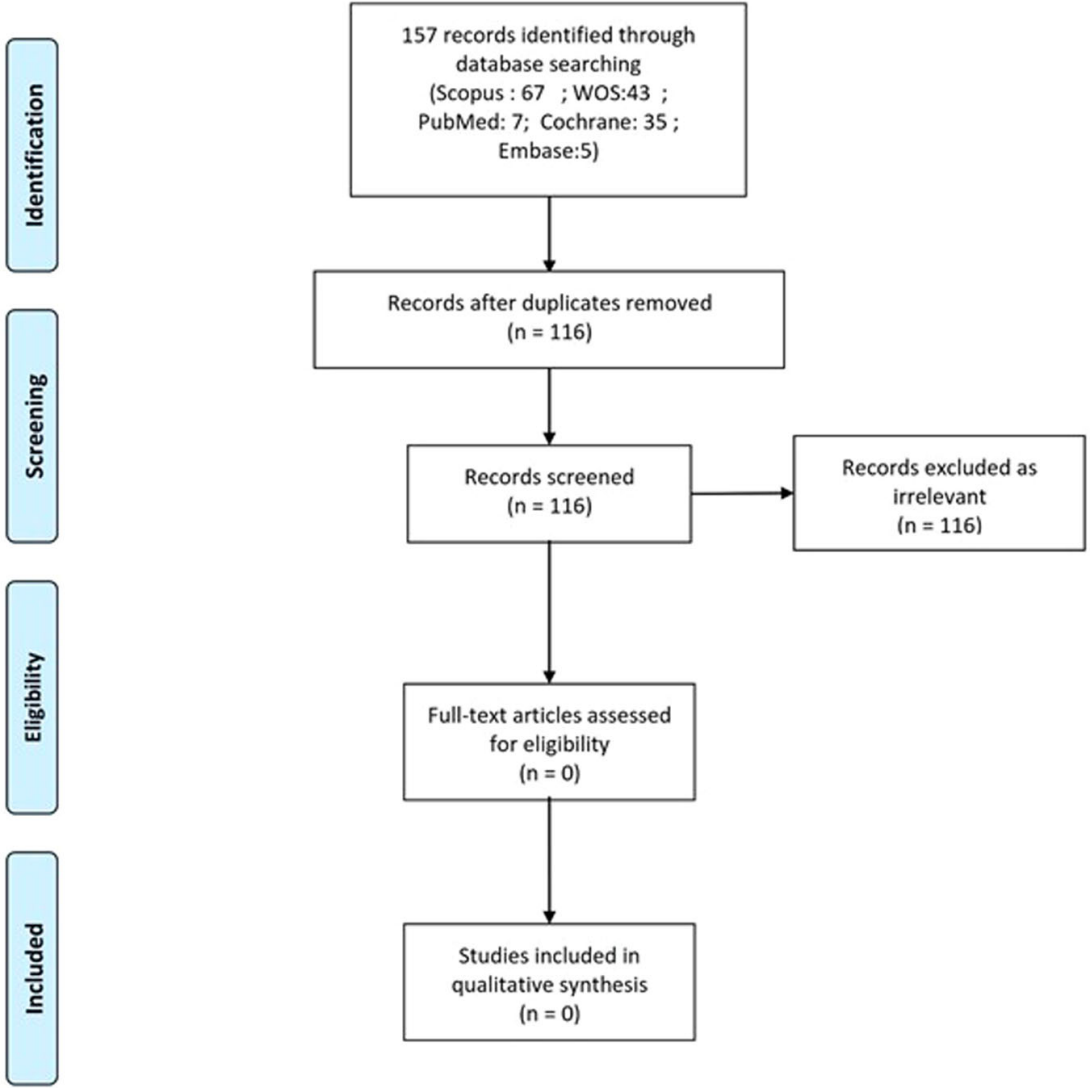
PRISMA flow diagram for study selection. The PRISMA flow diagram outlines the selection process for the studies included in this systematic review. Starting with 157 records identified from five major databases (Scopus, WOS, PubMed, Cochrane, Embase), the process involved removing duplicates, screening for relevance, and assessing full-text articles for eligibility. Ultimately, no studies met the criteria for inclusion, highlighting a significant research gap in the application of digital twins in dermatology. The diagram visually represents the filtering steps from identification to final inclusion, demonstrating the thorough methodology employed in this scoping review.

Although the use of digital twins for health has increasingly become popular^9^, a potential gap is identified in integrating this concept into dermatology. Despite searching five major digital libraries, PubMed, Scopus, Web of Science, Cochrane Library, and Embase, with a comprehensive set of keywords and lack of any language restrictions, as of 05 February 2024, we found no eligible relevant study after evaluating the included 157 papers (Fig. 2, and Supplementary file). Our findings revealed a significant gap in research focused on the application of digital twins in dermatology. This gap indicates that while digital twins have been successful in other areas of medicine, their potential in dermatology remains largely untapped.

## Digital twins in dermatology from clinical perspectives

The concept of digital twins in dermatology comes with two significant potentials from the perspective of clinical visits and trials. The former involves patients seeking medical care for different skin-related concerns, and through a thorough evaluation, dermatologists offer treatment recommendations and monitor their skin health over time. The latter is about research studies focused on developing and evaluating new treatments or procedures aimed at the skin condition through investigating safety, efficacy, and potential side effects. These perspectives can be significantly facilitated through the integration of digital twins, elaborated in the following.

### Potential in regular clinical visits

Imagine a dermatologist facing a patient with severe psoriasis, aiming to initiate biologic therapy to maximize efficacy while minimizing the risk of failure. Here, digital twins play a crucial role as a cutting-edge tool combining genetic data (deep phenotyping) with real-time environmental and lifestyle data (digital phenotyping) from IoT devices. In such a scenario, developing an appropriate physical-digital interface (cyber-physical systems) and using the analytical power of AI with such information can contribute. These comprehensive digital models allow dermatologists to simulate and evaluate the effects of various treatment options on the patient’s skin condition, potentially relevant mutations^27^, and associated comorbidities such as arthritis, fatty liver, hypertension, and insulin resistance.

Such models could extend beyond superficial analysis, offering insights into inflammation and other key biomarkers, as well as even skin microbiome, which is crucial for understanding the intricacies of the disease. The computational capability of digital twins to simulate the patient’s response to different therapies enables an accurate prediction of treatment outcomes. Consequently, the digital twin acts as a virtual testing environment, facilitating the selection of the most suitable biologic therapy that promises significant symptom relief, minimal side effects, and durable disease management, all tailored to the patient’s unique dermatological needs.

### Potential in clinical trials

In the context of clinical trials, the use of digital twins offers a novel approach to addressing ethical concerns associated with control arms, where patients might otherwise be deprived of effective treatments^28–31^. Traditional clinical trials necessitate the inclusion of a control group, often receiving a placebo or standard treatment, against which the efficacy of a new therapeutic intervention is measured. However, this model raises ethical dilemmas, especially in trials for conditions with severe morbidity or where effective treatments are already available. This issue is more highlighted with diseases like epidermolysis bullosa, in which randomized control trials (RCTs) may predispose the untreated control group on a placebo to a risk of highly aggressive squamous cell carcinoma.

Digital twins can revolutionize this aspect by providing a virtual control arm. With digital twins in place, a corresponding digital twin could be created for each participant in the treatment group of a clinical trial. When considering treatment options, the digital twin allows researchers to simulate the outcomes of various interventions, including the new treatment under investigation and the existing standard of care or placebo effect. In essence, the digital twin undergoes the “control” treatment in a virtual environment, eliminating the need to withhold potentially more effective treatments from any real patient. This method not only upholds the ethical principle of providing the best available care but also maintains the scientific rigor of clinical trials by allowing for a comparison between new interventions and the current standard without compromising patient well-being.

Moreover, as the trial progresses, real-time data from patients undergoing the actual treatment can be fed back into the digital twins, allowing for continuous refinement of the models and enabling adjustments to treatment protocols based on emerging data. This dynamic process enhances the predictive accuracy of the digital twins, making them even more valuable as virtual controls.

It is apparent that this innovative approach could be particularly transformative in dermatology, where the visual and symptomatic nature of skin conditions often requires immediate and effective treatment interventions. By integrating digital twins as virtual control arms in clinical trials, dermatology can advance its research methodology, ensuring patients receive the most effective treatments while generating robust data on new therapies’ efficacy and safety.

## Precision medicine in dermatology

Medical treatment and healthcare can significantly benefit from precision medicine (i.e., personalized medicine). It is an innovative approach that considers genetics, environment, and lifestyle differences for enhanced disease decision-making. Precision medicine in dermatology particularly customizes medical treatments for skin conditions based on individual patients’ characteristics. This treatment customization, however, is more complex than a simplistic binary approach, necessitating a progressive evolution toward more sophisticated models, benefiting from advancements in image processing with convoluted neural networks^32–34^ and multi-functioning skin biosensors^35–37^. Furthermore, innovative approaches from closely-related domains, their integration, and adaptability are valuable, e.g., analyzing skin temperature through a computer-simulated model through complex thermoregulation intended initially for energy and architectural research^38^. This model can shed new light on dermatologic applications if utilized correctly.

Additionally, novelties from domains other than medicine can be inspirational. Atashipour et al.^39^, from the mechanical engineering domain, have introduced a novel approach for predicting the material properties of soft tissues, including skin, by analyzing vibration data. They employed measured full-field displacements in a reverse-engineering algorithm to update models and create a digital twin of desired tissues, including skin, to examine its response to vibrations. Such methodologies could be pivotal in clinical dermatology for predicting the mechanical properties of multi-layered skin (including skin thickness) through resonance frequencies and comprehensive imaging techniques.

## Issues and future research direction

Implementing digital twins in healthcare raises significant data privacy and ethical issues. Dermatology, which often involves sensitive patient data related to skin conditions that can affect individuals’ appearance and psychological well-being, may face heightened concerns regarding such data’s secure and ethical use. This requires establishing clear guidelines and protocols for data privacy, consent, and security specific to using digital twins in dermatology as well as engaging patients in the conversation around data use.

Creating and maintaining digital twins demands substantial computational power and technical expertise. The field of dermatology may not yet have the infrastructure or the workforce adequately trained in these high-tech tools to implement digital twin technology effectively. This can become complicated further due to the existence of complex dermatological data. It is unlike conditions such as hypertension or diabetes, where disease parameters are relatively straightforward to quantify. Therefore, dermatological conditions involve complex visual data that require advanced image processing and pattern recognition technologies for effective digital twin modeling.

Digital twins can utilize electronic health records and other existing health data sources to enrich digital twins with real-world clinical data, enhancing their accuracy and utility in dermatological practice. This data integration should be accompanied by prioritizing, developing, and improving algorithms capable of processing and analyzing extensive dermatological data (e.g., images or telemetry data from medical-purpose IoT devices). However, advanced data pipelines and algorithms may incur costs and bring resource allocation challenges for the development and maintenance of digital twins within healthcare budgets. Conducting comprehensive cost-benefit analyses and exploring scalable, cost-effective technological solutions, such as “cloud computing,” “edge computing,” or “edge AI,” are vital steps to justify and manage the investment in digital twin technologies.

Developing digital twins not only requires close collaboration between clinicians, computer scientists, data analysts, and other experts, but also the interoperability challenges between disparate healthcare systems and the digital twin platform should be addressed. The seamless integration of diverse health data sources is crucial for accurately modeling digital twins. Thus, adopting universal healthcare data standards and developing adaptable middleware solutions can facilitate this integration, ensuring that digital twins can be effectively utilized across different healthcare settings. Moreover, the siloed nature of research and clinical practice may impede the interdisciplinary collaboration necessary to successfully integrate digital twins in dermatology. Hence, it necessitates interdisciplinary partnerships that encourage collaboration between dermatologists, computer scientists, data analysts, and patients to co-develop digital twin models that are clinically relevant and patient centric. It even requires developing specialized training programs for dermatologists and healthcare professionals on digital dermatology, including digital twin technology, focusing on its potential applications, benefits, and ethical considerations in dermatology.

Moreover, while rapidly evolving, digital twin technology is still considered nascent in many healthcare areas, including dermatology. Its application has been more prominent in sectors with a more extended digitization and data integration history, such as manufacturing and aerospace.There may also have been a historical bias towards investing in digital health innovations in fields with broader public health impacts, such as cardiology or neurology. This could inadvertently lead to dermatology being underrepresented in digital health research and development initiatives. Therefore, pilot projects can explore the feasibility and effectiveness of digital twins in managing specific dermatological conditions, such as dermatologic orphan diseases with limited populations for RCT.

Another critical issue is ensuring equitable access to digital twin technologies. There is a risk that such advanced treatments could be disproportionately available to those in technologically advanced or higher-income regions, exacerbating existing healthcare disparities. Addressing this requires innovative solutions, such as public-private partnerships to subsidize technology costs and utilizing widely accessible mobile technologies to democratize access to digital twin-based treatments^40^.

## Conclusion

The use of digital twins in dermatology promises enhanced diagnostic accuracy, optimized treatment plans, and improved patient monitoring by accommodating the unique complexities of skin conditions. The identified gap in utilizing digital twins application emphasizes the need for research to address challenges, including the intricate nature of skin diseases, ethical and privacy concerns, and the necessity for specialized algorithms. Overcoming these barriers through interdisciplinary efforts and focused research is essential for realizing digital twins’ potential in dermatology, highlighting the need for a tailored approach to dermatological care as personalized as the patients themselves. Hence, dermatology can advance toward a future where personalized and predictive care is envisioned and realized through the power of digital twins.

### Supplementary information

Supplemetary File
