# Supplementary material for: Digital twins in dermatology, current status, and the road ahead
Source: NPJ Digit Med. 2024 Aug 26;7:228. doi: 10.1038/s41746-024-01220-7 (PMC11347578; doi:10.1038/s41746-024-01220-7)
Supplement: Supplementary file 1 — Supplemetary File [file 41746_2024_1220_MOESM1_ESM.pdf]

Our systematic review was meticulously designed to dissect and synthesize the burgeoning field of digital twin technology within dermatology. By casting a wide net over the existing literature, we aimed to unearth the scope of digital twin applications across dermatologic care, understanding their influence on diagnosis, treatment, and patient management strategies.

Formulating the Research Question: Central to our investigation was the question, "How is digital twin technology currently being applied within dermatology, and what future directions does this suggest for personalized dermatologic care?". This overarching query guided our systematic exploration, necessitating a methodological approach that could comprehensively capture the multifaceted nature of digital twin technology's integration into dermatology. The systematic review was rigorously structured, adhering to the PRISMA-ScR guidelines[1] to ensure a transparent and replicable research process.

Developing a Robust Search Strategy: Our search strategy was meticulously crafted, combining extensive keywords pertinent to digital twins and dermatologic applications (Table 1). We scoured five principal electronic databases: Web of Sciences, Scopus, PubMed, Embase, and Cochrane Library, up to 05th February 2024, aiming to capture a comprehensive field snapshot.

Rigorous Study Selection Process: Post-duplication elimination, we embarked on a detailed screening process. Reviewers meticulously evaluated titles and abstracts against a set of pre-defined inclusion and exclusion criteria designed to sieve through the literature for studies that elucidate the application of digital twins in dermatology.

- **Inclusion Criteria:**
  - Research articles that investigate the application of digital twin technology in dermatological settings.
  - Publications that are available in any language ensure a comprehensive global perspective.
- **Exclusion Criteria:**
  - Studies that focus on the broader applications of digital twin technology outside the specific context of dermatology.
  - Research that mentions digital twin technology but does not explore its application or implications within dermatological practice.
  - Duplicate publications, conference abstracts, and articles from non-peer-reviewed sources which may compromise the review's scientific rigor.

Discrepancies were resolved through consensus, underscoring the collaborative nature of our review process. The inclusion criteria were deliberately broad to encompass all studies that shed light on digital twin technology within dermatologic contexts without any language restriction.

Comprehensive Data Extraction and Synthesis:

A data extraction form was designed to collect nuanced details from each study, ranging from the geographical origins and publication year to the study design and key findings related to digital

twin applications in dermatology. This granular approach enables a rich data synthesis, painting a vivid portrait of how digital twins are being utilized to enhance dermatologic care. We undertook a comprehensive data extraction process for this systematic review to collect detailed information from each included study. The extraction details were systematically organized around several key dimensions to ensure a thorough evaluation and synthesis of the literature on digital twin technology in dermatology. The following data were meticulously considered to be recorded:

- **Author(s) and Publication Year:** Aimed at identifying key contributors and temporal trends in digital twin research within dermatology.
- **First Author Country:** To assess the geographical diversity and concentration of research efforts.
- **Target Issue or Overall Aim:** Focused on the specific dermatological issues addressed or the overarching goals of the research.
- **Research Concept:** The foundational theories or hypotheses driving the application of digital twins in dermatological studies.
- **Modeling Methods or Twinning Elements Used:** Detailed technical methodologies and the specific elements of digital twinning employed in the studies.
- **Dermatological Conditions Studied:** The range of skin conditions explored through digital twin technology.
- **Diagnostic and Treatment Approaches:** The diagnostic methods and treatment strategies examined within the context of digital twins, including the outcomes reported.

Elevated Quality Assessment:
Adopting the Oxford Centre for Evidence-based Medicine tool[2] , we aimed to critically appraise the quality of studies that could have been included, ensuring our review's findings were based on a robust evidence base.

Ethical Considerations Reiterated:
Reflecting on the ethical scope of our review, we emphasize that our literature synthesis did not require new data collection from human participants, thus negating the need for ethical approval.

Dissemination:
Our multi-faceted dissemination strategy aims to share our findings across peer-reviewed journals and academic conferences. This approach is intended to engage a wide audience, including clinicians, researchers, and policymakers, to foster dialogue on the potential of digital twins in dermatology and encourage further research and application in this promising area.

Supplementary Table 1 – search query in databases

Databases	Search in	Key words

PubMed	*(Title/Abstract for all options) including mesh words*	(“digital twin” OR “digital replica*”) AND ("Skin" or "Derm" or "Cutaneous" or “dermatitis” or “eczema” or "Hyperpigmentation" or "Porphyria cutanea tarda" or "Pruritus" or "Xerosis" or "Palmar erythema" or "Histiocytosis" or "Alopecia" or "hair loss" or "Alopecia areata" or "Leukonychia" or "Onycholysis" or "nail" or "Scleroderma" or "Psoriasis" or "Calciphylaxis" or "Eruptive xanthomas" or "Xanthelasma" or "Vesiculobullous eruptions" or "Lichen planus" or "Pityriasis rubra pilaris" or "Palmoplantar hyperkeratosis" or "Erythema multiforme" or "Erythrokeratodermia variabilis" or "Angiokeratoma corporis diffusum" or "Granuloma annulare" or "Eruptive angiomas" or "Prurigo nodularis" or "Beau lines" or "Poikiloderma" or "Cutaneous calcinosis" or "Seborrheic keratoses" or "Hirsutism" or "Hypertrichosis" or "Skin fragility" or "Yellow skin discoloration" or "Raynaud" or "Periungual telangiectasia" or "Eccrine gland necrosis" or "Sarcoid-like granulomas" or "Nephrogenic systemic fibrosis" or "Pseudo-Kaposi sarcoma" or "Vitiligo" or "Focal palmoplantar keratoderma" or "Erythroderma" or "Pyoderma gangrenosum" or "Cutaneous polyarteritis nodosa" or "Splinter hemorrhages" or "Sweet's syndrome" or "Dermatomyositis" or "Livedo reticularis" or "Urticaria pigmentosa" or "Bullous pemphigoid" or "Angiomatosis" or "Xeroderma Pigmentosum" or "Skin cancer" or "Skin Neoplasms" or "Squamous Cell Carcinoma"or "Melanoma" or "Merkel Cell Carcinoma" or "Basal Cell Carcinoma")

Scopus	*Title, Abstract and Keywords for all options*	(“digital twin” OR “digital replica*”) AND ("Skin" or "Derm" or "Cutaneous" or “dermatitis” or “eczema” or "Hyperpigmentation" or "Porphyria cutanea tarda" or "Pruritus" or "Xerosis" or "Palmar erythema" or "Histiocytosis" or "Alopecia" or "hair loss" or "Alopecia areata" or "Leukonychia" or "Onycholysis" or "nail" or "Scleroderma" or "Psoriasis" or "Calciphylaxis" or "Eruptive xanthomas" or "Xanthelasma" or "Vesiculobullous eruptions" or "Lichen planus" or "Pityriasis rubra pilaris" or "Palmoplantar hyperkeratosis" or "Erythema multiforme" or "Erythrokeratodermia variabilis" or "Angiokeratoma corporis diffusum" or "Granuloma annulare" or "Eruptive angiomas" or "Prurigo nodularis" or "Beau lines" or "Poikiloderma" or "Cutaneous calcinosis" or "Seborrheic keratoses" or "Hirsutism" or "Hypertrichosis" or "Skin fragility" or "Yellow skin discoloration" or "Raynaud" or "Periungual telangiectasia" or "Eccrine gland necrosis" or "Sarcoid-like granulomas" or "Nephrogenic systemic fibrosis" or "Pseudo-Kaposi sarcoma" or "Vitiligo" or "Focal palmoplantar keratoderma" or "Erythroderma" or

		"Pyoderma gangrenosum" or "Cutaneous polyarteritis nodosa" or "Splinter hemorrhages" or "Sweet's syndrome" or "Dermatomyositis" or "Livedo reticularis" or "Urticaria pigmentosa" or "Bullous pemphigoid" or "Angiomatosis" or "Xeroderma Pigmentosum" or "Skin cancer" or "Skin Neoplasms" or "Squamous Cell Carcinoma"or "Melanoma" or "Merkel Cell Carcinoma" or "Basal Cell Carcinoma")
Web of Sciences	Abstract, title	(“digital twin” OR “digital replica*”) AND ("Skin" or "Derm" or "Cutaneous" or “dermatitis” or “eczema” or "Hyperpigmentation" or "Porphyria cutanea tarda" or "Pruritus" or "Xerosis" or "Palmar erythema" or "Histiocytosis" or "Alopecia" or "hair loss" or "Alopecia areata" or "Leukonychia" or "Onycholysis" or "nail" or "Scleroderma" or "Psoriasis" or "Calciphylaxis" or "Eruptive xanthomas" or "Xanthelasma" or "Vesiculobullous eruptions" or "Lichen planus" or "Pityriasis rubra pilaris" or "Palmoplantar hyperkeratosis" or "Erythema multiforme" or "Erythrokeratodermia variabilis" or "Angiokeratoma corporis diffusum" or "Granuloma annulare" or "Eruptive angiomas" or "Prurigo nodularis" or "Beau lines" or "Poikiloderma" or "Cutaneous calcinosis" or "Seborrheic keratoses" or "Hirsutism" or "Hypertrichosis" or "Skin fragility" or "Yellow skin discoloration" or "Raynaud" or "Periungual telangiectasia" or "Eccrine gland necrosis" or "Sarcoid-like granulomas" or "Nephrogenic systemic fibrosis" or "Pseudo-Kaposi sarcoma" or "Vitiligo" or "Focal palmoplantar keratoderma" or "Erythroderma" or "Pyoderma gangrenosum" or "Cutaneous

		polyarteritis nodosa" or "Splinter hemorrhages" or "Sweet's syndrome" or "Dermatomyositis" or "Livedo reticularis" or "Urticaria pigmentosa" or "Bullous pemphigoid" or "Angiomatosis" or "Xeroderma Pigmentosum" or "Skin cancer" or "Skin Neoplasms" or "Squamous Cell Carcinoma"or "Melanoma" or "Merkel Cell Carcinoma" or "Basal Cell Carcinoma")
Cochrane	*abstract, title and keywords for all options*	(“digital twin” OR “digital replica*”) AND ("Skin" or "Derm" or "Cutaneous" or “dermatitis” or “eczema” or "Hyperpigmentation" or "Porphyria cutanea tarda" or "Pruritus" or "Xerosis" or "Palmar erythema" or "Histiocytosis" or "Alopecia" or "hair loss" or "Alopecia areata" or "Leukonychia" or "Onycholysis" or "nail" or "Scleroderma" or "Psoriasis" or "Calciphylaxis" or "Eruptive xanthomas" or "Xanthelasma" or "Vesiculobullous eruptions" or "Lichen planus" or "Pityriasis rubra pilaris" or "Palmoplantar hyperkeratosis" or "Erythema multiforme" or "Erythrokeratodermia variabilis" or "Angiokeratoma corporis diffusum" or "Granuloma annulare" or "Eruptive angiomas" or "Prurigo nodularis" or "Beau lines" or "Poikiloderma" or "Cutaneous calcinosis" or "Seborrheic keratoses" or "Hirsutism" or "Hypertrichosis" or "Skin fragility" or "Yellow skin discoloration" or "Raynaud" or "Periungual telangiectasia" or "Eccrine gland necrosis" or "Sarcoid-like granulomas" or "Nephrogenic systemic fibrosis" or "Pseudo-Kaposi sarcoma" or "Vitiligo" or "Focal palmoplantar keratoderma" or "Erythroderma" or "Pyoderma gangrenosum" or "Cutaneous polyarteritis nodosa" or "Splinter

		hemorrhages" or "Sweet's syndrome" or "Dermatomyositis" or "Livedo reticularis" or "Urticaria pigmentosa" or "Bullous pemphigoid" or "Angiomatosis" or "Xeroderma Pigmentosum" or "Skin cancer" or "Skin Neoplasms" or "Squamous Cell Carcinoma"or "Melanoma" or "Merkel Cell Carcinoma" or "Basal Cell Carcinoma")
Embase	*Abstract, title, and keywords for all option*	(“digital twin” OR “digital replica*”) AND ("Skin" or "Derm" or "Cutaneous" or “dermatitis” or “eczema” or "Hyperpigmentation" or "Porphyria cutanea tarda" or "Pruritus" or "Xerosis" or "Palmar erythema" or "Histiocytosis" or "Alopecia" or "hair loss" or "Alopecia areata" or "Leukonychia" or "Onycholysis" or "nail" or "Scleroderma" or "Psoriasis" or "Calciphylaxis" or "Eruptive xanthomas" or "Xanthelasma" or "Vesiculobullous eruptions" or "Lichen planus" or "Pityriasis rubra pilaris" or "Palmoplantar hyperkeratosis" or "Erythema multiforme" or "Erythrokeratodermia variabilis" or "Angiokeratoma corporis diffusum" or "Granuloma annulare" or "Eruptive angiomas" or "Prurigo nodularis" or "Beau lines" or "Poikiloderma" or "Cutaneous calcinosis" or "Seborrheic keratoses" or "Hirsutism" or "Hypertrichosis" or "Skin fragility" or "Yellow skin discoloration" or "Raynaud" or "Periungual telangiectasia" or "Eccrine gland necrosis" or "Sarcoid-like granulomas" or "Nephrogenic systemic fibrosis" or "Pseudo-Kaposi sarcoma" or "Vitiligo" or "Focal palmoplantar keratoderma" or "Erythroderma" or "Pyoderma gangrenosum" or "Cutaneous polyarteritis nodosa" or "Splinter

		hemorrhages" or "Sweet's syndrome" or "Dermatomyositis" or "Livedo reticularis" or "Urticaria pigmentosa" or "Bullous pemphigoid" or "Angiomatosis" or "Xeroderma Pigmentosum" or "Skin cancer" or "Skin Neoplasms" or "Squamous Cell Carcinoma"or "Melanoma" or "Merkel Cell Carcinoma" or "Basal Cell Carcinoma")
Web of Sciences	*(Title/Abstract for all options)*	("digital twin" OR "digital replica*") AND ("Skin" or "Derm" or "Cutaneous" or "dermatitis" or "eczema" or "Hyperpigmentation" or "Porphyria cutanea tarda" or "Pruritus" or "Xerosis" or "Palmar erythema" or "Histiocytosis" or "Alopecia" or "hair loss" or "Alopecia areata" or "Leukonychia" or "Onycholysis" or "nail" or "Scleroderma" or "Psoriasis" or "Calciphylaxis" or "Eruptive xanthomas" or "Xanthelasma" or "Vesiculobullous eruptions" or "Lichen planus" or "Pityriasis rubra pilaris" or "Palmoplantar hyperkeratosis" or "Erythema multiforme" or "Erythrokeratodermia variabilis" or "Angiokeratoma corporis diffusum" or "Granuloma annulare" or "Eruptive angiomas" or "Prurigo nodularis" or "Beau lines" or "Poikiloderma" or "Cutaneous calcinosis" or "Seborrheic keratoses" or "Hirsutism" or "Hypertrichosis" or "Skin fragility" or "Yellow skin discoloration" or "Raynaud" or "Periungual telangiectasia" or "Eccrine gland necrosis" or "Sarcoid-like granulomas" or "Nephrogenic systemic fibrosis" or "Pseudo-Kaposi sarcoma" or "Vitiligo" or "Focal palmoplantar keratoderma" or "Erythroderma" or "Pyoderma gangrenosum" or "Cutaneous polyarteritis nodosa" or "Splinter

		hemorrhages" or "Sweet's syndrome" or "Dermatomyositis" or "Livedo reticularis" or "Urticaria pigmentosa" or "Bullous pemphigoid" or "Angiomatosis" or "Xeroderma Pigmentosum" or "Skin cancer" or "Skin Neoplasms" or "Squamous Cell Carcinoma"or "Melanoma" or "Merkel Cell Carcinoma" or "Basal Cell Carcinoma")

Supplementary References:

1 McGowan, J. *et al.* Reporting scoping reviews—PRISMA ScR extension. *Journal of clinical epidemiology* **123**, 177-179 (2020).

2 Howick, J. *et al.* The 2011 Oxford CEBM evidence levels of evidence (introductory document). *Oxford Center for Evidence Based Medicine* (2011).
